# Discontinuous video recording of biopsies in the context of an integral third degree teaching program

**DOI:** 10.1186/1746-1596-3-S1-S8

**Published:** 2008-07-15

**Authors:** José Ernesto Moro Rodríguez

**Affiliations:** 1 Universidad Rey Juan Carlos. Pathology Anatomy Area. Alcorcón, Madrid, Spain

## Abstract

**Background:**

The application of Information and Communication Technologies (ICTs) in the field of university education can play an important role in order to fulfil the objectives of the European Space for Higher Education (ESHE). In the absence of an infrastructure that allows virtual preparations, the high definition video may become an alternative for many PC users and telepathology enthusiasts.

**Methods:**

We have begun a video biopsy discontinuous recording pilot programme, also known as interval cinematography, in order to give our students pathological images to gather documentary evidence to follow their clinical cases. We have a working post available with a ZEISS Axioshop 2 microscope connected to a JVC digital camera with a double output for video, a PVM_14N1MDE Sony Triniton colour monitor and a LG RH199 HDD-DVD recorder.

**Results:**

Pathologists can easily produce videos lasting between 45" and 5' according to the case. Later those videos are assessed all together and given to the student and/or the doctor who requested the case study.

**Conclusion:**

Better follow-up studies of the cases can be achieved using Discontinuous video recording of biopsies. This approach has also clinical benefits since it contributes to the enrichment of integral patient care.

## Introduction

The application of Information and Communication Technologies (ICTs) in the field of university education can play an important role in order to fulfil the objectives of the European Space for Higher Education (ESHE). ICTs can be a valid tool when considering lifelong learning as part of higher education. At the Berlin conference (2003:4), the term lifelong learning was defined as the "continuous learning process that allows individuals, from childhood to old age, to acquire and update knowledge, skills and competences in the different periods of their life and in a variety of learning contexts, formal as well as non-formal, therefore maximizing their personal development, job opportunities and fostering their active participation in a democratic society".

The Rey Juan Carlos University, the youngest of the six public universities in Madrid, was founded in 1996. The faculty of Health Sciences is located in the district of Alcorcón, in the south of Madrid, with a population of 160,000 inhabitants. It began its teaching activity in April 1997 starting with a three-year degree in Physiotherapy and Occupational Therapy. One year later, a three-year degree in Nursing began and, in the academic year 2003–2004, a five-year degree in Odontology. A five-year degree in Medicine is expected to commence in the academic year 2008–2009.

The five-year degree in Odontology aims to train odontologists to act in different areas of dental clinics, as well as in health prevention and promotion. Studies leading to a five-year degree in Odontology are structured in two cycles: a two and a three-year cycle respectively, with a total of 329.5 credits. In the first cycle, the basic subjects are concentrated, while the second cycle includes more specific subjects. An important part of the credits are practical lessons in laboratories and clinics with attention to patients. The availability of a great number of laboratories with experimental facilities, as well as care clinics, allows students, supervised by university teachers, to carry out practical lessons and treat patients.

Fifth-year Odontology students teach the subjects Integrated Odontological Clinic for Adults, Integrated Odontological Clinic for Children and Integrated Odontological Clinic for Special Patients. In addition, in a third cycle, students have the option of developing their skills doing a Master in Implant and Oral Surgery. It is in those three above-mentioned subjects in their degree and in the Master where the Histology and Pathology Areas have developed an information flow system with the objective of collaborating with the rest of the teachers, in order to inform as to the diagnostic means of the infectious, traumatological, tumoral and dysembrioplastic or dysmorphic processes of the oral-facial area, so that the student can carry out a diagnosis, prognosis and a proposal for treatment.

In the absence of an infrastructure that allows virtual preparations, the high definition video may become an alternative for many PC users and telepathology enthusiasts. No matter if we watch high definition TV at home or a DVD while travelling, we always prefer high quality images. The new video formats that are being launched into the market (H.264, HMV-HD and MPEG-2) meet that demand and allow us to watch high definition films on our computers. The high definition video (HDV) offers a practical functioning scheme easily accepted by both consumers and manufacturers. All things considered, this might be a good solution since it is not as costly as the professional HD production systems (Sony HDCam and Panasonic DVCPro HD) and it offers a reasonable quality and fidelity similar to the DV video (MiniDV, DVCam or DVCPro).

## Materials and methods

In this context, we have begun a video biopsy discontinuous recording pilot programme, also known as interval cinematography, in order to give our students pathological images to gather documentary evidence to follow their clinical cases. We have a working post available with a ZEISS Axioshop 2 microscope connected to a JVC digital camera with a double output for video, a PVM_14N1MDE Sony Triniton colour monitor and a LG RH199 HDD-DVD recorder.

## Results

Our work flow is as follows: once the anatomopathological study request of a biopsy coming from the Odontology University Clinic has been registered, the material is processed according to the usual routine and assessed in the microscope by the pathologist. Together with microscopic assessment, the pathologist carries out a discontinuous video recording on the fields he considers to be the most interesting for the final diagnosis. Those videos last between 45" and 5' according to the case. Later those videos are assessed all together and given to the student and/or the doctor who requested the case study.

## Conclusion

Through this procedure we have been able to carry out better follow-up studies of the cases and we have found the point of view of the clinic beneficial as it contributes to the enrichment of integral patient care.

